# Insight into Oily Sludge Treatment by High-Temperature Bioaugmentation: Petroleum Components Degradation and Microbial Community Regulation

**DOI:** 10.3390/microorganisms14071470

**Published:** 2026-07-03

**Authors:** Xinge Fu, Jin Li, Qinghong Wang, Xuankai Zeng, Hui Zuo, Juntao Jiang, Rui Zhang, Peng Zhao, Muhammad Usman, Mohamed Gamal El-Din, Chunmao Chen

**Affiliations:** 1 Shandong Key Laboratory of Green Electricity & Hydrogen Science and Technology, School of Chemical Engineering, Shandong Institute of Petroleum and Chemical Technology, Dongying 257061, China; 2 State Key Laboratory of Heavy Oil Processing, China University of Petroleum-Beijing, Beijing 102249, China; 3 State Key Laboratory of Microbial Diversity & Innovative Utilisation, Institute of Microbiology, Chinese Academy of Sciences, Beijing 100110, China; 4 Department of Civil and Environmental Engineering, University of Alberta, Edmonton, AB T6G 1H9, Canada; musman4@ualberta.ca

**Keywords:** oily sludge, high-temperature bioaugmentation, petroleum components, thermophilic bacteria, microbial community

## Abstract

Thermochemical washing of oily sludge is a highly effective strategy for resource recovery. However, the lingering petroleum components within washed sludge still pose considerable environmental pollution risks. In this study, high-temperature bioaugmentation technology was used to promote the removal of petroleum components, and the potential of this technology in the deep treatment of washed oily sludge was investigated, specifically petroleum component degradation and microbial community regulation. The results showed that the petroleum degradation performance was greatly enhanced by the introduction of thermophilic bacteria combined with 200 mg/kg of biosurfactant (T2 group). Under these conditions, the petroleum and moisture contents were successfully reduced to 19.6 g/kg and 4%, respectively. In particular, the contents of n-alkanes and polycyclic aromatic hydrocarbons in the T2 group decreased significantly to 1098.5 and 494.3 mg/kg, respectively, relative to the blank group (4428.5 and 850.7 mg/kg). High-throughput sequencing results indicated that exogenous thermophilic bacteria (*Ureibacillus* and *Bacillus*) could rapidly emerge as the dominant genera in the system. In addition, microbial association network and functional analyses revealed that high-temperature bioaugmentation shifted microbial interactions from competition to cooperation, as evidenced by increased positive correlation ratios and modularity values, as well as functional diversification from two modules in the blank group to four modules in the biofortified treatments, thereby enhancing synergistic degradation capabilities for hydrocarbons. This study provides a newly developed approach to oily sludge treatment that simultaneously achieves reduction and harmlessness.

## 1. Introduction

The petrochemical industry produces a large amount of oily sludge in the process of crude oil extraction, storage and refining [1]. The global annual output is more than 60 million tons, and the cumulative stock has exceeded 1 billion tons [2]. Oily sludge, a type of bulk hazardous waste, consists of crude oil, water, and solid components such as sand, soil, and mud. In particular, toxic and harmful components, such as heavy metals, hydrocarbons, the benzene series, and pathogenic microorganisms trapped in oily sludge can potentially threaten the atmosphere, water, and soil environments [3]. Recently, increasing attention has been paid to the treatment of oily sludge. Pyrolysis technology effectively eliminates petroleum components and recovers valuable byproducts, such as pyrolysis oil and gas; nevertheless, the drying process significantly increases energy consumption [4]. Thermochemical washing technology is capable of recovering crude oil, but the residual crude oil content (>2%) fails to meet the emission standards [5]. Biodegradation is typically considered an eco-friendly treatment technology for oily sludge. However, its widespread adoption is hindered by various limitations, including the low activity of degrading microorganisms, and a significant impact from environmental factors. Therefore, it is crucial to explore efficient and compatible microbiota, along with advanced regulatory techniques to overcome these challenges.

Bioaugmentation technology refers to the method of improving the removal efficiency of target pollutants by adding strains with specific functions to the natural environment during the biological treatment process. This technology can enhance the activity of petroleum-degrading microorganisms and improve the bioavailability of oily sludge, thereby significantly boosting biodegradation [6]. A single petroleum-degrading strain cannot completely degrade the intricate composition of oily sludge, leading to inadequate treatment efficiency [7]. However, the synergistic and thorough degradation of petroleum contaminants can be accomplished by assembling a community of petroleum-degrading strains [8]. Nie et al. [9] highlighted that *Bacillus* sp. strain NYGH exhibited a preference for degrading alkanes, whereas *Planococcus citreus* strain T2 preferentially degraded aromatics. Upon combining the two strains, the degradation rate of petroleum in oily sludge increased to 78%, representing an increase ranging from 17.5% to 34.4% compared to the single bacterial system. Chand et al. [10] obtained a petroleum degradation rate of approximately 53.1% during the biodegradation of heavy petroleum-contaminated soil by using microbial community composed of *Rhodococcus ruber*, *Bacillus* sp., and *Bacillus cereus*. Notably, more than 90% of short-chain n-alkanes and 12% of the asphaltenes could be effectively degraded within 90 d. In short, the construction of a petroleum-degrading microbial community is an effective strategy for enhancing the treatment efficiency of oily sludge.

Meanwhile, some petroleum-degrading bacteria have been proven to produce surfactants during their metabolic processes, which significantly improves the efficiency of petroleum degradation. Biosurfactants exhibit excellent emulsifying properties because of their hydrophilic and hydrophobic ends [11]. Therefore, they can reduce interfacial tension and promote the emulsification of petroleum droplets, dispersing insoluble organic pollutants such as hydrocarbons, polycyclic aromatic hydrocarbons (PAHs), and polychlorinated biphenyls into the aqueous phase, thereby increasing the affinity between microbial cells and hydrophobic organics and enhancing biodegradation efficiency [12]. Common biosurfactants include glycolipids, lipopeptides, phospholipids, etc. Compared with chemical surfactants, they have higher biodegradability, better environmental compatibility and lower toxicity, and thus possess excellent application prospects in petroleum pollution remediation. At present, it has been widely reported that biosurfactants can effectively promote petroleum degradation. For example, Urum et al. [13] found that rhamnolipid can remove up to 80% of crude oil, with an effect comparable to that of chemical surfactant sodium dodecyl sulfate. The lipopeptide biosurfactant produced by bacterial strain Ar70C7-2 isolated from deep-sea reservoirs in Brazil exhibits good emulsifying properties and interfacial activity and could remove about 30% of petroleum [14]. Meanwhile, many surfactant-producing bacteria (e.g., *Bacillus*, and *Pseudomonas*) are widely used in the field of petroleum pollution remediation. For instance, Atakpa et al. [15] discovered that *Acinetobacter* sp. Y2, which is capable of producing surfactants, promote petroleum degradation. Consequently, the screening and identification of high-performance bacteria that possess the dual capability of degrading petroleum and producing surfactants is of immense significance for the treatment of oily sludge [16].

Temperature plays an important role in the microbial degradation of hydrocarbons. Although the degradation of petroleum hydrocarbons by mesophilic bacteria has been widely studied, research on the degradation of thermophilic bacteria is still lacking. An increase in temperature can decrease the viscosity of oily sludge and improve the solubilization of organic compounds, which is conducive to biodegradation. In addition, enzymes from thermophilic bacteria are more resistant to denaturation and grow faster. Therefore, high-temperature bioaugmentation may be a potential method to enhance the reduction and harmless treatment of oily sludge. Thermophilic hydrocarbon degraders (e.g., *Thermus* and *Bacillus*) have been isolated from natural extreme environments and have shown potential for oil remediation [17,18]. Meanwhile, high-temperature conditions can alter the composition of microbial communities, affect interspecific interactions, and alter microbial activity and metabolic functions [19]. In addition, high temperatures can cause changes in environmental variables such as humidity and dissolved oxygen, thereby further regulating the structural composition and function of microbial communities. Furthermore, high- temperature conditions have a significant effect on the bacterial degradation of hydrocarbons. Enzyme activity is regulated by temperature, and enzyme catalysis is involved in almost all chemical conversion reactions within cells, thus affecting the efficiency of microbial degradation. However, the survival ability of microorganisms is limited under high-temperature conditions, which can reduce biodegradation efficiency. It is worth noting that the addition of external biosurfactants can significantly improve the bioavailability of hydrocarbons, thereby effectively promoting the proliferation of degrading bacteria, and can still significantly enhance the degradation activity of microorganisms in high-temperature environments [19]. At present, applied research on high-temperature bioaugmentation by thermophilic bacteria inoculation in oily sludge treatment is very limited. Specifically, it is not clear what the oil degradation characteristics of exogenous thermophilic bacteria in the sludge matrix are or how the succession of the microbial community is carried out. In biological treatment systems of oily sludge, the research on the enhancement of oil degradation by thermophilic bacteria inoculation combined with biosurfactant addition is even more limited.

In this study, a high-temperature bioaugmentation system for washed oily sludge was constructed by inoculating a thermophilic petroleum-degrading flora combined with a biosurfactant. The petroleum degradation performance, compositional changes in petroleum components, and microbial community succession were analyzed during the bioaugmentation process. The research aims were threefold: (1) to evaluate the petroleum degradation performance of the proposed high-temperature bioaugmentation system; (2) to analyze changes in the content of n-alkanes and PAHs during the process; and (3) to reveal the succession of the microbial communities. This study predicts that the developed high-temperature bioaugmentation technology will achieve good petroleum hydrocarbon removal efficiency and drive favorable microbial community succession, thereby providing technical support for the high-temperature bioaugmentation treatment of oily sludge.

## 2. Materials and Methods

### 2.1. The Source of Oily Sludge and Sawdust

The oily sludge sample was obtained from the primary oil separator of a petrochemical company in Beijing, China (38°23′24″ N, 116°29′3″ E). The sludge had been pretreated by thermochemical washing before sampling, and the collected sample was the residual solid phase after the washing process. The collected sample was transferred to a clean and sealed container, sealed immediately to minimize volatilization losses, and transported back to the laboratory under ice bath conditions for subsequent biodegradation experiments. The basic physicochemical properties of the thermochemically washed samples are presented in Table S1.

Sawdust, purchased from a local market (in the Changping District, Beijing, China), serves as the carrier of the petroleum-degrading bacteria. The sawdust was ground to below 20 mesh size and sterilized before use.

### 2.2. The Source and Preparation of Thermophilic Microbial Agents

The microbial agent used for high-temperature bioaugmentation was a thermophilic petroleum-degrading consortium derived from the collected oily sludge sample. A 10 g portion of oily sludge was transferred into a conical flask containing 250 mL of sterile water. After shaking at 50 °C for 3 h, 5 mL of the supernatant was inoculated into 100 mL of a crude oil inorganic salt medium containing 1 g/L of crude oil, followed by incubation at 150 r/min and 50 °C for 5 d. After five successive enrichment rounds, gradient sample diluents (10^−1^, 10^−2^, 10^−3^, 10^−4^, 10^−5^) were spread onto LB agar plates, distributed evenly, and placed in an incubator at 50 °C for 2–3 days until clear colonies had grown. Three bacterial strains with relatively high petroleum degradation capacities were selected. Subsequently, the strains were identified via PCR amplification and sequencing of the 16S rDNA gene. The three strains were *Bacillus velezensis* AQ-1, *Ureibacillus thermosphaericus* AQ-3 and *Brevibacillus agri* AQ-23. The morphologies of the three petroleum-degrading strains are shown in Figure S1.

Crude oil inorganic salt medium includes 1 g/L KH_2_PO_4_, 0.5 g/L K_2_HPO_4_, 1.3 g/L (NH_4_)_2_SO_4_, 10 g/L NaCl, 0.2 g/L MgSO_4_, 0.1 g/L CaCl_2_, and 0.01 g/L of FeSO_4_. The pH was adjusted to 7.0 using 1 M NaOH and 1 M HCl, and 0.5% (*m*/*v*) crude oil was added to the medium after sterilization at 121 °C for 20 min.

The three bacterial colonies were cultured in an LB liquid culture at 50 °C with shaking for activation and were used to prepare a bacterial suspension once cultivation reached the end of the logarithmic growth phase. The bacterial solution was centrifuged (8000 r/min, 5 min) to discard the supernatant, resuspended with sterile saline, and repeated twice. Finally, the bacterial suspension OD_600_ was adjusted to 0.8 with sterile saline for use. The three bacterial suspensions were mixed in equal proportions and then mixed with sawdust and placed in a biochemical incubator. The temperature was slowly increased to 60 °C within 2 h to accelerate water evaporation and allow the bacterial cells to fully adsorb onto the sawdust to obtain an immobilized petroleum-degrading bacterial agent.

### 2.3. Performance Evaluation of Petroleum-Degrading Strains

The bacterial suspension was added to the crude oil inorganic salt medium with an inoculation amount of 5% (*v*/*v*), and placed in a shaker at 50 °C for 7 d. The contents of petroleum and n-alkanes (C_10_-C_40_) in mediums were measured. Three strains of thermophilic petroleum-degrading bacteria were inoculated in LB medium for activation. After the medium was turbid, the fermentation medium was inoculated with 5% (*v*/*v*) for 3 days. After the culture, the fermentation broth was centrifuged at 8000 r/min for 10 min to obtain the supernatant for detecting whether the strain had surfactant production performance. The performance tests of surfactant production include droplet collapse, diameter of oil discharge ring, and emulsification index, and the determination method is based on the report by Thirumurugan et al. [20]. The purification of biosurfactant was based on the method of Joy [21]. The specific steps were as follows: The fermentation broth was centrifuged at 10,000 r/min for 10 min to obtain the fermentation supernatant, and the pH value of the supernatant was adjusted to 2.0 with 6 mol/L hydrochloric acid. After standing in the refrigerator at 4 °C for 24 h, the precipitate was obtained by centrifugation at 10,000 r/min for 10 min, and the centrifuged supernatant was extracted three times with the same amount of ethyl acetate. The organic phase was combined, and the solvent was evaporated on a rotary evaporator. The solute and precipitate were combined and washed three times with deionized water and dried in a freeze dryer to obtain a biosurfactant solid powder. The type of biosurfactant was identified by thin-layer chromatography (TLC) and Fourier transform infrared spectroscopy (FT-IR), and the critical micelle concentration (CMC) was determined to optimize the dosages of surfactant. CMC was determined according to the method of Datta et al. [22], and TLC was determined according to the method of Zouari et al. [23]. FT-IR was determined by the potassium bromide tabletting method (Magna-IR 560 ESP, America).

Fermentation medium includes 1 g/L KH_2_PO_4_, 0.5 g/L K_2_HPO_4_, 1.5 g/L peptone, 10 g/L NaCl, 0.2 g/L MgSO_4_, 0.1 g/L CaCl_2_, 0.01 g/L FeSO_4_, and 1.0% (*m*/*v*) n-hexadecane. The pH was adjusted to 7.0 using 1 M NaOH and 1 M HCl. Sterilization was performed at 121 °C for 20 min.

### 2.4. Optimization of Biosurfactant Dosage for High-Temperature Bioaugmentation

The concentration of biosurfactant was used as the only variable to determine the optimal amount of biosurfactant added to maximize petroleum degradation efficiency. Five treatment levels were set. Biodegradation experiments were carried out by adding 0, 50, 100, 200 and 800 mg/kg biosurfactant produced by *Brevibacillus agri* AQ-23 to the treatment system, labeled as Blank, T50, T100, T200 and T800, respectively. All treatment groups were carried out under the same conditions. Firstly, microbial agents were prepared by adding 50 mL bacterial suspension into a beaker containing 25 g sawdust and culturing in a high-temperature biochemical incubator (60 °C) for 2 h to accelerate the immobilization of microorganisms on sawdust. Then, 500 g of oily sludge sample was mixed evenly with the prepared microbial agent. Finally, five levels of biosurfactant were added respectively, and the moisture content was adjusted to 45% by sterile water. The mixture was continuously cultured at 60 °C for 7 days. The pile was turned once daily to ensure oxygen circulation. The petroleum content of the sludge during the biodegradation test was determined on days 0, 1, 3, 5, and 7. All experiments were conducted in triplicate, and the results were averaged.

### 2.5. High-Temperature Bioaugmentation Treatment of Oily Sludge in Bioreactors

Three groups of experiments were conducted to evaluate the performance of bioaugmentation for the treatment of oily sludge. A total of 5 kg of oily sludge sample was added to three bioreactors: the blank group did not include the natural attenuation of microbial agents, the T1 group included the bioaugmentation of thermophilic microbial agents, and the T2 group added 200 mg/kg biosurfactant produced by *Brevibacillus agri* AQ-23 in addition to thermophilic microbial agents. The thermophilic microbial agents consisted of 500 mL of bacterial suspension and 250 g of sawdust. All of the materials were thoroughly mixed in a bioreactor, then the initial moisture content was adjusted to 45%. The temperature of the reactor was controlled at 60 °C using an oil bath, and a paddle stirrer was positioned inside to blend the materials once a day. The bioreactor setup is illustrated in Figure S2. During the experimental period, the petroleum and moisture contents were measured. At the end of the experiment, petroleum components (n-alkanes and PAHs), related enzyme activities (dehydrogenase and polyphenol oxidase), and microbial communities were analyzed.

### 2.6. Analytical Methods

#### 2.6.1. Determination of Petroleum Components

The content of petroleum components is calculated based on the dry weight of oily sludge. According to National Environmental Protection Standards of China (HJ 1051-2019) [24], the petroleum content was measured using an infrared oil analyzer (OIL480, Beijing Zhongchuang Instrument Technology Co., Ltd., Beijing, China). According to China’s national environmental protection standards HJ 1021-2019 [25] and HJ 805-2016 [26], the contents of n-alkane (C_10_-C_40_) and PAHs were determined by Gas chromatography-mass spectrometry (7890B-5977B, Agilent, Santa Clara, CA, USA). Based on different chain lengths, n-alkanes are divided into short-chain (C_10_-C_14_), medium-chain (C_15_-C_30_), and long-chain (C_31_-C_40_) alkanes. The degradation rate of petroleum components was calculated according to Formula (1).

(1) $$\omega \left(\%\right)=\frac{c_{0}-c_{1}}{C_{0}}\times 100$$

where ω is the degradation rate, *C*_0_ stands for the initial content of petroleum components, and *C*_1_ is the residual content.

#### 2.6.2. Enzyme Activity and High-Throughput Sequencing Analysis

The activities of dehydrogenase (DHA) and polyphenol oxidase (PPO) were measured using corresponding colorimetric assay kits (Shanghai Jining Industrial Co., Ltd., Shanghai, China), with 2,3,5-Triphenyl Tetrazolium Chloride (37 °C, 24 h) and pyrogallol (30 °C, 1 h) as substrates, respectively. The total genomic DNA of the microbial community was extracted according to the instructions of the FastDNA ^®^ Spin Kit (MP Biomedicals, Santa Ana, CA, USA). The quality of the extracted genomic DNA was detected by 1% agarose gel electrophoresis, and the concentration and purity of DNA were determined by NanoDrop2000 (Thermo Scientific, Waltham, MA, USA). The V3–V4 variable region of the 16S rRNA gene was amplified by PCR using the upstream primer 338F (5′-ACTCCTACGGGAGGCAGCAG-3′) and the downstream primer 806R (5′-GGACTACHVGGGTWTCTAAT-3′). A Pro Taq, 20 μL reaction system was used in the PCR test. The PCR products were recovered using 2% agarose gel, purified using a DNA gel recovery and purification kit (PCR Clean-Up Kit C01-10000, Yuhua Biotechnology Co., Ltd., Shanghai, China), and quantified using Qubit 4.0 (Thermo Fisher Scientific, Waltham, MA, USA). The NEXTFLEX Rapid DNA-Seq Kit (Bioo Scientific, Austin, TX, USA) was used to construct a library of purified PCR products. Sequencing was performed using the Illumina Miseq PE300 platform (Shanghai Meiji Biomedical Technology Co., Ltd., Shanghai, China). Quality control of paired-end raw sequencing reads was performed using fastp software (version 0.20.0) and assembly was conducted using FLASH software (version 1.2.7). Based on default parameters, denoising of the optimized sequences after quality control and assembly was carried out using the DADA2 (version 2024.10.0) plugin in the Qiime2 (version 2024.10) pipeline. The sequences obtained after DADA2 denoising were designated as ASVs (amplicon sequence variants). To minimize the influence of sequencing depth on diversity data analysis, all samples were rarefied to the minimum sample sequence count. After rarefaction, the average sequence coverage per sample still reached 99.99%. Taxonomic classification of ASVs was performed using the Naive Bayes classifier in Qiime2 based on the Silva 16S rRNA gene database.

### 2.7. Data Analysis

One-way ANOVA followed by Duncan’s multiple range test was used for multiple comparisons, with Duncan chosen for its higher power in pairwise comparisons among all treatments. A significance level of *p* < 0.05 was applied, using SPSS (version 21.0). Alpha diversity indices were calculated using mothur software (version 1.30.2). Stacked bar plots generated with R (version 3.3.1) were used to identify the most abundant bacterial communities at the phylum and genus levels. To explore the correlations of petroleum component contents and key enzyme activities with the dominant genera (top 50), a heatmap was generated using the vegan package (version 2.4-0) in R (version 3.3.1). The functions of metabolism and degradation in the bioaugmentation process were predicted using the PICRUSt2 (version 2.2.0). Co-occurrence network analysis was carried out to elucidate the co-occurrence pattern of microbial communities in each treatment group. Based on Spearman correlation |r| > 0.6, *p* < 0.05, dominant species (top 50) were selected for correlation network analysis. The relationship between the dominant genus (top 30) and metabolic and degradation functions was further analyzed by co-occurrence network. Using Gephi (version 0.10) software was used for topology analysis, modular analysis and visualization.

## 3. Results

### 3.1. Performance Evaluation of Exogenous Thermophilic Bacteria

**Figure 1:**
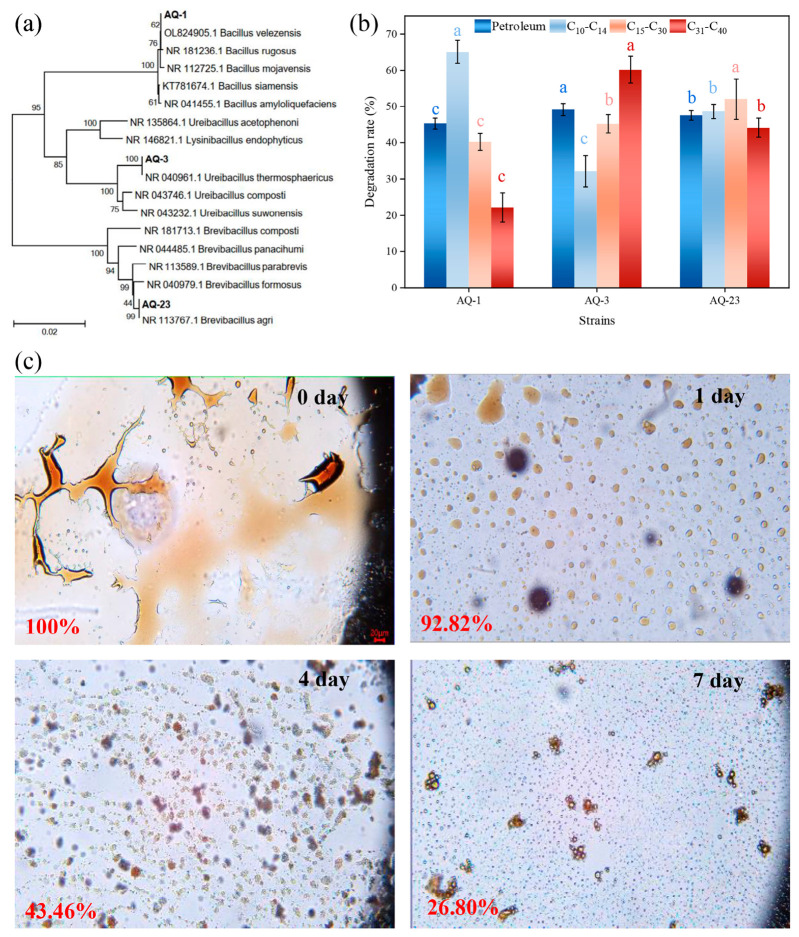
Characteristic of thermophilic microbial agents. (**a**) Evolutionary development tree of each strain; (**b**) Degradation rate of petroleum and petroleum hydrocarbons by strains; (**c**) The state change in crude oil in the degradation process observed by microscope. Different letters indicate significant differences at the *p* < 0.05 level (Duncan’s test).

As shown in Figure 1a, AQ-1 (accession No. PQ721805), AQ-3 (accession No. PQ721806), and AQ-23 (accession No. PQ721807) represent three distinct petroleum-degrading strains isolated from oily sludge that have been identified as belonging to *Bacillus velezensis*, *Ureibacillus thermosphaericus* and *Brevibacillus agri*, respectively. As shown in Figure S1, *Bacillus velezensis* AQ-1 is short rod-shaped, while *Ureibacillus thermosphaericus* AQ-3 and *Brevibacillus agri* AQ-23 are long rod-shaped. The three strains demonstrated remarkable petroleum degradation capabilities within 7 d, achieving degradation rates exceeding 45% (Figure 1b). However, distinct preferences and efficiencies were observed for the degradation of n-alkanes with varying chain lengths. *Bacillus velezensis* AQ-1 showed excellent degradation performance toward short-chain n-alkanes (ranging from C_10_ to C_14_). The degradation rate for these alkanes surpassed 60%, indicating their strong ability to decompose shorter chain n-alkanes. *Ureibacillus thermosphaericus* AQ-3 demonstrated a particular ability to degrade long-chain n-alkanes (spanning from C_31_ to C_40_). With a degradation rate exceeding 55%, this strain is well-suited for breaking down the more complex, longer chain n-alkanes. *Brevibacillus agri* AQ-23 exhibited a more balanced degradation profile across various petroleum components, indicating that it could effectively degrade both short- and long-chain alkanes.

As shown in Figure 1c, the flora constructed with the three strains in equal proportions exhibited excellent petroleum degradation capabilities (73.2%). The petroleum was emulsified and dispersed into small droplets, and the color gradually faded. These observations indicate that the effective biodegradation of petroleum may be related to the production of biosurfactants during the microorganism metabolic process. Therefore, the capacities of the three strains to produce biosurfactants were further investigated. As shown in Table S2, *Brevibacillus agri* AQ-23 could induce significant droplet collapse and form a large oil drainage ring with a diameter of 5.0 cm, confirming its ability to produce surfactants. Its emulsification index (55%) was greater than 50%, indicating that *Brevibacillus agri* AQ-23 was a surfactant-producing strain with superior emulsifying properties. The characterization of the biosurfactant produced by *Brevibacillus agri* AQ-23 was determined by TLC and FT-IR (Figure S3). The silica gel plate sprayed with the ninhydrin-acetone reagent exhibited a distinct purple-red color, indicating that the surfactant produced by *Brevibacillus agri* AQ-23 belongs to the lipopeptide surfactant [27]. The presence of CO–N and N–H bonds also confirmed its classification as a lipopeptide surfactant [28]. The CMC of this biosurfactant was 100 mg/L, and its production yield reached 3.03 g/L.

However, the limited addition amount of *Brevibacillus agri* AQ-23 in the oily sludge biodegradation system makes it difficult to produce sufficient biosurfactants for achieving an optimal petroleum emulsification effect in the short term. An additional biosurfactant was further added to promote petroleum removal from the oily sludge. The biosurfactant dosage for the bioaugmentation treatment was optimized (Figure S4). The results showed that the addition of biosurfactant efficiently improved petroleum degradation. When the dosage of the biosurfactant reached 200 mg/kg, the petroleum content decreased from 61.8 to 20.3 g/kg, achieving a 67.2% petroleum degradation rate. After further increasing the dosage of the biosurfactant, the increase in the petroleum degradation rate was insignificant. Thus, in the high-temperature bioaugmentation system, the additional biosurfactant dosage was set at 200 mg/kg.

### 3.2. The Enhanced Performance of Oily Sludge Degradation by High-Temperature Bioaugmentation

#### 3.2.1. Changes in Petroleum Content and Moisture Content

**Figure 2:**
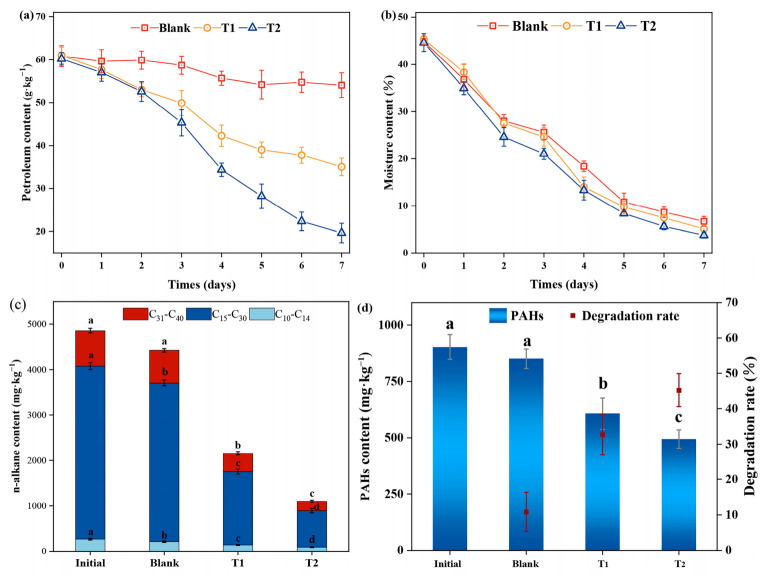
The content changes in petroleum (**a**); moisture (**b**); n-alkanes (**c**) and PAHs (**d**) during the bioaugmentation process. Different letters indicate significant differences at the *p* < 0.05 level (Duncan’s test).

After thermochemical washing, oily sludge was subjected to high-temperature bioaugmentation. Figure 2a,b shows the changes in petroleum and moisture contents in the three biodegradation groups. In the blank group, the petroleum content decreased slightly from 60.83 to 54.08 g/kg. The decrease in petroleum content was significant when using bioaugmentation by thermophilic microbial agents (T1), or thermophilic microbial agents and a biosurfactant (T2), with residual contents of 35.02 g/kg and 19.62 g/kg after 7 days of biodegradation. Compared with the T1 group, the oil degradation rates in the T2 group increased by 25.37%. Eventually, the petroleum content of the T2 group (19.6 g/kg) was below 20 g/kg, which met the petroleum content control standards of SY/T 7301-2016 [29] and significantly reduced the risk of pollution. The moisture content in the three biodegradation groups exhibited minimal differences and rapidly declined in the system under high-temperature conditions. By the sixth day, the moisture content had decreased to below 10%, and the moisture content in the T2 group decreased faster than that in the other groups. The degradation of petroleum-containing components and the evaporation of water are important factors for reducing the mass of oily sludge. As shown in Table S3, before and after high-temperature treatment, the reduction degrees of the oily sludge treatment system in the three treatment groups were 50.01% (CK), 52.45% (T1), and 56.05% (T2), demonstrating that high-temperature treatment can achieve more than 50% reduction in the oily sludge treatment system after 7 days, and the reduction degree after bioaugmentation treatment was greater than that after natural attenuation treatment.

#### 3.2.2. The Degradation of Petroleum Components

Figure 2c shows the changes in n-alkane contents with different chain lengths in each biodegradation group. The initial contents of short-chain, medium-chain and long-chain n-alkanes in the oily sludge were 262.5, 3810.0 and 788.0 mg/kg, respectively, with medium- and long-chain n-alkanes being the primary components. After seven days of biodegradation treatment, the removal of medium-chain n-alkanes was the most significant in each group, with contents being reduced to 3500 (blank), 1615 (T1), and 801 mg/kg (T2). Generally, long-chain n-alkanes exhibit low bioavailability owing to their strong hydrophobicity and low water solubility, rendering them challenging pollutants for biodegradation [30]. Consequently, in the blank group (719 mg/kg), the long-chain alkanes were almost unchanged. However, the addition of thermophilic microbial agents accelerated the decomposition of long-chain n-alkanes, reducing the content to 408 mg/kg. The presence of surfactants can increase the affinity of microorganisms for long-chain alkanes [31]. Therefore, in the T2 group, the long-chain n-alkanes content was further degraded to 205 mg/kg. The contents of long-chain n-alkanes were 719 mg/kg (blank group), 408 mg/kg (T1), and 205 mg/kg (T2). It can be seen that the medium-chain n-alkanes were effectively degraded after bioaugmentation, and the degradation rates of the T1 and T2 groups were 57.61% and 78.98%, respectively. Similarly, the degradation rates of long-chain n-alkanes in the T1 and T2 groups were 48.22% and 73.98%, respectively, indicating that these components were also effectively removed.

In addition, PAHs are resistant to degradation by microorganisms because of their structural stability, high hydrophobicity, and complex molecular structures [32]. They have been classified as priority pollutants because of their mutagenic and carcinogenic effects on humans and aquatic ecosystems [33]. The removal performance of PAHs was significantly different among the various treatment groups. In the blank group, the concentration of PAHs was reduced from 902.7 to 850.7 mg/kg, indicating that indigenous microorganisms alone are insufficient for effectively degrading PAHs (Figure 2d). However, compared with the blank group, the PAHs in the T1 and T2 groups were significantly reduced, with total contents decreasing to 607.0 and 494.3 mg/kg, corresponding to degradation rates of 32.7% and 45.2%, respectively. This finding indicates that high-temperature bioaugmentation treatment significantly enhances the removal of PAHs. Moreover, the addition of biosurfactants further and significantly improved the removal of PAHs. Compared with previous studies, this bioaugmentation technology effectively promoted the degradation of n-alkanes and PAHs [34,35]. The volatilization potential of petroleum hydrocarbons was further examined. The oily sludge was sterilized under an ultraviolet lamp and incubated at 60 °C for 7 days. The results (Figure S5) showed that the volatilization rates of C_10_-C_40_ and PAHs were 0.79% and 0.37%, respectively, indicating that the volatilization of petroleum hydrocarbons was limited during the process of high-temperature bioaugmentation. Therefore, it can be inferred that biodegradation plays a dominant role in the decomposition of petroleum hydrocarbon components in the high-temperature bioaugmentation of thermochemically washed oily sludge.

### 3.3. Microbial Community Succession During High Temperature Bioaugmentation Process

#### 3.3.1. Analysis of Enzyme Activity

**Figure 3:**
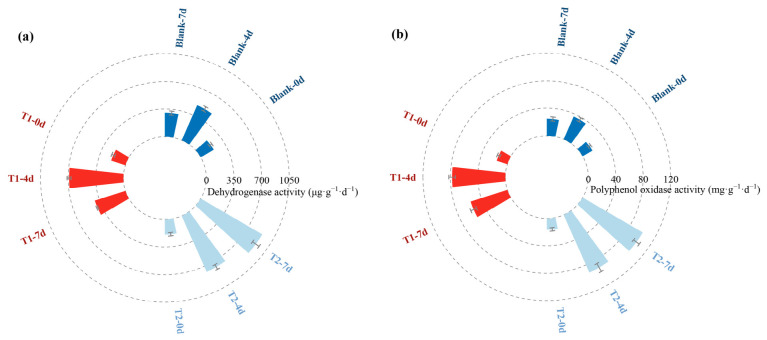
Changes in dehydrogenase activity (**a**); polyphenol oxidase activity (**b**) during the bioaugmentation process.

The changes in enzyme activities reflect the activity of the microorganisms in the system. As shown in Figure 3, the dehydrogenase and polyphenol oxidase activities in the bioaugmentation groups (T1 and T2) were higher than those in the blank group, indicating that bioaugmentation can effectively improve the metabolic activity of degrading bacteria and enhance the degradation of petroleum. The enzyme activities in the blank and T1 groups showed an initial increase followed by a decrease, which is consistent with the degradation trend of petroleum. The petroleum content decreased rapidly during the initial stage (0–4 d) and tended to stabilize after 4 d. In the later stages of degradation, the decrease in enzyme activity was caused by nutrient deficiency. However, the enzyme activities in the T2 group continued to increase during the later stage.

#### 3.3.2. Analysis of Microbial Community Structure and Main Functional Bacteria

The effect of bioaugmentation on the microbial community was determined using 16s rRNA to compare its diversity, structure, and functions. The ACE, Chao, and Sobs indices were reduced in all groups, indicating that the diversity of the microbial community and species richness decreased during biodegradation (Table S4). This was probably due to the inability of some bacteria to adapt to high-temperature environments.

**Figure 4:**
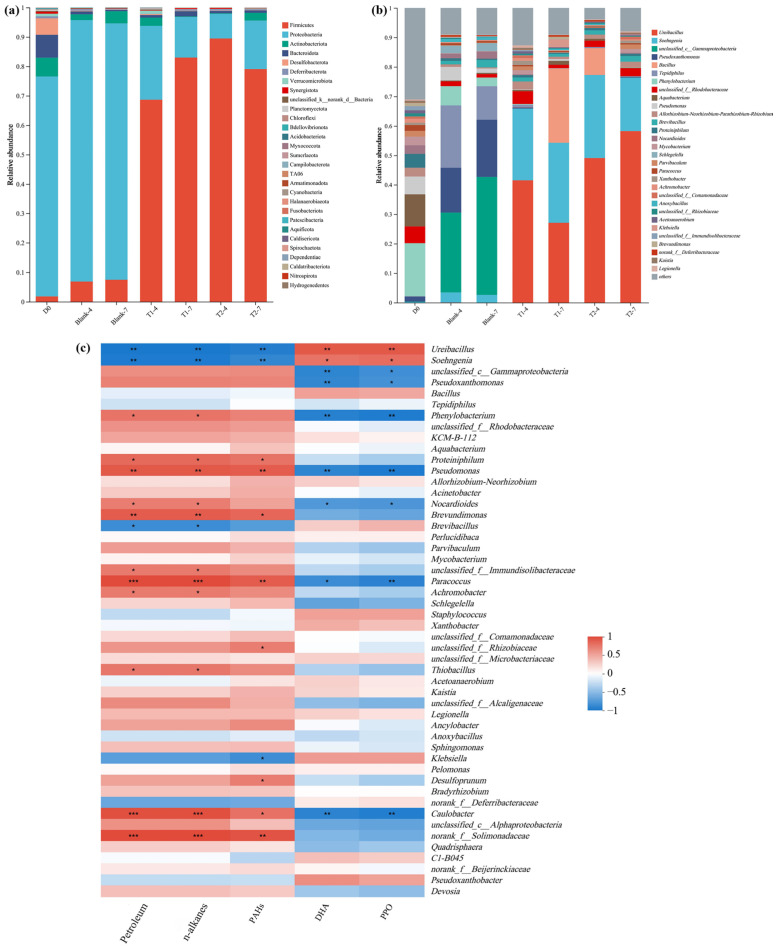
Changes in microbial community structure at phylum level (**a**); and genus level (**b**); and correlation analysis of petroleum components, key enzyme activity and main bacteria genera (**c**), * 0.01 < *p* < 0.05, ** 0.001 < *p* < 0.01, *** *p* < 0.001.

Figure 4a shows the distribution of microbial communities in the different treatment groups at the phylum level. It was found that the dominant species in the oily sludge was Proteobacteria, accounting for 74.8%. After biodegradation, Proteobacteria was still the dominant phylum in the blank group (88.9%). However, bioaugmentation caused significant changes in the microbial community structure. Firmicutes replaced Proteobacteria as the primary dominant phylum in T1 and T2 group, accounting for 68.7% and 89.7%, respectively. This is because the three strains of thermophilic microbial agents introduced belong to Firmicutes, which is capable of thriving and multiplying abundantly under high-temperature conditions. The enrichment of Firmicutes was beneficial for petroleum degradation, and similar results have also been reported in a previous study [36].

The composition of the bacterial communities at the genus level differed between the blank and bioaugmentation groups (Figure 4b). *Phenylobacterium* (18%) was the primary dominant genus within the initial microbial community, while the predominant genera of *Gammaproteobacteria* (27.1~40.1%), *Pseudoxanthomonas* (15.3~19.4%), and *Tepidiphilus* (11.3~21.2%) were the members of the blank group during the high-temperature treatment. This indicates that these genera possess certain capabilities for high-temperature resistance and petroleum degradation. However, due to the addition of exogenous bacteria (*Bacillus*, *Ureibacillus* and *Brevibacillus*), the microbial community structure in the bioaugmentation groups changed significantly compared with the blank group (Figure S6). *Ureibacillus* grew rapidly in both T1 and T2 groups and swiftly became the dominant bacteria with a relative abundance of 41.5–49.1% (on the 4th day) in the biodegradation system. However, the emulsification of petroleum provided a more readily available carbon source for microorganisms, resulting in a significant difference (*p* < 0.05) in *Ureibacillus* abundance between the two bioaugmented treatment groups on the 7th day. Consequently, *Ureibacillus* continued to increase in the T2 group (58.3%), while it decreased in the T1 group (27.1%). On the 4th day, the abundance of *Bacillus* also showed a significant difference between the two bioaugmented groups (*p* < 0.05). The relative abundance of *Bacillus* in the T2 group (9%) was much higher than that in the T1 group (0.4%), indicating that the addition of the surfactant could promote the enrichment of this exogenous bacterium. Unexpectedly, the abundance of *Brevibacillus* (1–2%) remained very low in both groups, with no significant difference. Differences in the abundance of exogenous bacteria may be caused by hydrocarbon suppression, nutrient availability, and temperature [37]. The relative abundance of *Soehngenia* ranked second only to *Ureibacillus* in both bioaugmentation groups, which has been confirmed to actively participate in and significantly promote the degradation of petroleum [38].

The correlation between the primary bacteria and the degradation rates of petroleum components, as well as the activities of key enzymes, is shown in Figure 4c. The relative abundances of the dominant genera *Ureibacillus* and *Soehngenia* showed significant positive correlations with dehydrogenase and polyphenol oxidase activities, as well as significant negative correlations with the contents of petroleum, n-alkanes, and PAHs. These correlations suggest that these two genera may be involved in the degradation of petroleum components and may be associated with the secretion of key enzymes. Additionally, the relative abundance of *Klebsiella* showed a significant negative correlation with the PAH content, suggesting that this genus may have the potential to decompose PAHs. *Brevibacillus* also plays an important role in petroleum and n-alkane degradation. Furthermore, it is evident that the exogenous bacteria *Bacillus* and *Brevibacillus* are also involved in the secretion of these key enzymes. Previous studies have reported that *Bacillus* and *Brevibacillus* can produce various enzymes, enabling them to utilize a range of substrates for the production of biosurfactants [39,40]. This capability significantly improves the affinity between microorganisms and substrates and enhances the bioavailability of oily sludge [41,42,43,44].

#### 3.3.3. Microbial Association Network Analysis

**Figure 5:**
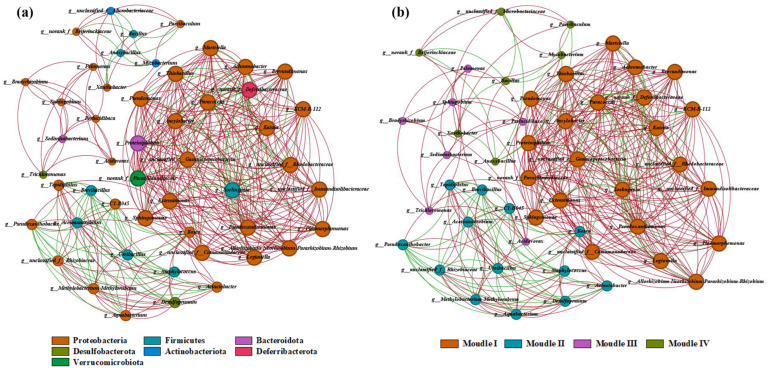
Microbial co-occurrence network map of the T2 group colored by Phylum level (**a**) and by module (**b**). The circular nodes represent bacterial genera, and the thickness of node lines is proportional to the value of Pearson correlation coefficient.

Microbial association network analysis is a powerful tool for exploring potential interactions among diverse bacterial groups in complex communities. Figure S7a (Blank group) and Figure S8a (T1 group) and Figure 5a (T2 group) depict the microbial association network diagrams based on the phylum level in the different treatment groups. These diagrams revealed that the microbial network was constructed from numerous bacterial genera that were highly interconnected within distinct bacterial clusters. These genera may occupy different positions and play diverse functional roles in the system. The nodes belonging to Proteobacteria and Firmicutes in the three treatment groups accounted for 77.55% (blank group), 86% (T1 group), and 84% (T2 group) of all nodes, respectively. The topological parameters of the network diagrams are listed in Table S5. The average degree (AD) reflects the complexity of the network. The AD values of the three treatment groups were 29.89 (blank group), 18.72 (T1 group) and 14.92 (T2 group), indicating that high-temperature bioaugmentation reduced the complexity of the bacterial network [45]. However, the proportion of positive correlations among different genera (red line) in the T1 (66.88%) and T2 (76.41%) groups increased compared to the blank group (63.39%), which could be attributed to the enhanced interspecific cooperation induced by the introduction of exogenous microorganisms.

The modularity (MD) values for the blank, T1, and T2 groups were 0.16, 0.33, and 0.49, respectively. These figures revealed that the blank group had the simplest modular structure, implying that the majority of microorganisms were interconnected in a small world [46]. In contrast, the T1 and T2 groups had distinct modular characteristics. The microbial collinearity networks of the three groups are shown in Figure S7b (Blank group), Figure S8b (T1 group), and Figure 5b (T2 group), where the different modules are labeled with different colors. There were two modules in the blank group, while four modules were observed in the T1 and T2 groups.

#### 3.3.4. Functional Analysis of Dominant Genera

**Figure 6:**
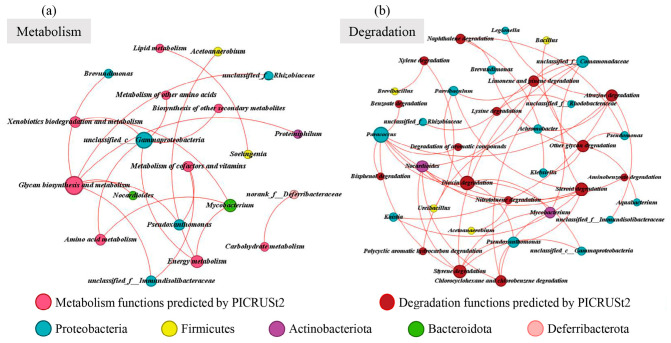
Network map based on positive correlations between dominant genera (top 30) and function profiles related to metabolism (**a**) and degradation (**b**).

In this study, PICRUSt2 was used to predict the degradation and metabolism functions of the microorganisms, and a co-occurrence network between the dominant bacteria and these functions in the high-temperature bioaugmentation system was constructed (Figure 6). The results showed that the majority of the dominant bacteria in the system were closely associated with metabolism and degradation functions, especially energy metabolism and styrene, polycyclic aromatic hydrocarbon, naphthalene, and xylene degradation.

Notably, several key bacteria were predicted to exhibit multiple metabolic functions. For instance, *Paracoccus* exhibits various functions related to the degradation of styrene, aromatic compounds, benzoate, and aminobenzoate. *Nocardioides* was significantly correlated with styrene, steroid, *chlorocyclohexane*, and chlorobenzene degradation. Mycobacterium possesses significant potential for various applications, exhibiting a robust correlation with energy, amino acid, and xenobiotic metabolism, and is closely related to the biodegradation of xenobiotics, styrene, *chlorocyclohexane*, chlorobenzene, dioxin, limonene, and pinene. Additionally, the exogenous thermophilic microbial agents (*Ureibacillus* and *Brevibacillus*) were significantly correlated with xylene degradation, indicating that those exogenous bacteria mainly play a role in degrading xylene. In addition, several dominant genera showed a potential ability to degrade petroleum components, such as *Klebsiella*, *Brevundimonas*, *Achromobacter*, *Pseudoxanthomonas*, *Nocardioides*, *Mycobacterium*, and *Paracoccus*. These key genera could represent important functional microorganisms in the high-temperature bioaugmentation treatment system of oily sludge.

## 4. Discussion

The three thermophilic bacterial strains isolated in this study exhibited complementary petroleum degradation characteristics, with *Bacillus velezensis* AQ-1 preferentially degrading short-chain alkanes, *Ureibacillus thermosphaericus* AQ-3 targeting long-chain alkanes, and *Brevibacillus agri* AQ-23 showing a broad-spectrum degradation capability. This functional complementarity explains why the constructed consortium achieved a superior degradation rate (73.2%) compared to the individual strains (>45%). Notably, *Brevibacillus agri* AQ-23 was identified as a lipopeptide biosurfactant producer with a CMC of 100 mg/L and a yield of 3.03 g/L, values that compare favorably with those of previously reported biosurfactants, suggesting its potential for application [47,48,49]. The observed emulsification and dispersion of petroleum during degradation further support the role of biosurfactants in facilitating hydrocarbon bioavailability. However, the limited amount of this strain in the system necessitated exogenous biosurfactant supplementation, with 200 mg/kg identified as the optimal dosage. The decline in petroleum degradation efficiency caused by excessive dosing of biosurfactants can be attributed to the formation of micelles that create a physical barrier on cell membranes, thereby reducing the affinity between microorganisms and petroleum components [50]. This underscores the importance of dosage optimization in bioaugmentation strategies.

In this study, the high-temperature bioaugmentation approach demonstrated remarkable efficiency, achieving 67.2% petroleum degradation within 7 days. This was attributed to the effective incorporation of biosurfactants. Specifically, biosurfactants facilitate emulsification by reducing the interfacial tension between petroleum and water, thereby enhancing the accessibility of petroleum to microorganisms for degradation [51]. Previous studies have demonstrated that a petroleum removal rate of only 39.3% was obtained while employing bioremediation technology to treat petroleum-contaminated soil for 141 d [52]. On the other hand, previous researchers enhanced the biological treatment of petroleum-contaminated soil through the addition of rhamnolipid, resulting in a petroleum biodegradation rate of 19.2% within 90 days [53]. This result reveals that the high-temperature bioaugmentation approach substantially outperforms conventional bioremediation methods. Moreover, the accelerated moisture reduction in biosurfactant-amended treatments suggests that enhanced hydrocarbon degradation may promote sludge dewatering, which is an additional benefit for waste volume reduction.

Microbial community analysis revealed that bioaugmentation fundamentally reshaped the indigenous microbiome, with Firmicutes replacing Proteobacteria as the dominant phylum due to the successful establishment of the introduced thermophilic strains. *Ureibacillus* emerged as the keystone genus, its abundance strongly correlating with both enzyme activities (dehydrogenase and polyphenol oxidase) and hydrocarbon degradation rates, confirming its central role in the process. The sustained enzyme activity in the T2 group, contrasting with the decline observed in the T1 and blank groups, suggests that biosurfactant addition helped maintain strong enzyme activity in the high-temperature bioaugmentation system even during the later stages when moisture became limiting. Admittedly, under very low moisture conditions, dehydrogenase and polyphenol oxidase activities do not necessarily demonstrate active microbial metabolism and may also reflect residual or extracellular enzymatic activity. The enrichment of *Soehngenia*, which can promote the degradation of petroleum components, and the correlation between *Klebsiella* and the removal of PAHs proved that bioaugmentation also stimulated the activity of indigenous degraders. In particular, although *Soehngenia* is typically an anaerobe, its high abundance in this turned reactor might be speculatively attributed to the formation of oxygen-limited microenvironments. The continuous rotation created dead zones with limited mixing, and the addition of biosurfactants promoted the aggregation of organic particles and microbial flocs, which further restricted oxygen diffusion inside the aggregates. These conditions could potentially have allowed *Soehngenia* to proliferate. The introduced *Bacillus* and *Brevibacillus* contributed to petroleum degradation, but their relative abundances were low. In particular, the relative abundance of *Brevibacillus* did not exceed 2% during the entire treatment process. This supports the concept that functional redundancy and community balance, rather than simple enrichment of specific degraders, are key to effective bioremediation. In addition, previous studies have demonstrated that the enrichment of petroleum-degrading bacteria is not a prerequisite for accelerating petroleum degradation [54]. Instead, the balance and diversity of the microbial community structure are essential for petroleum remediation [54]. Therefore, it can be speculated that *Bacillus* and *Brevibacillus* mainly promote petroleum degradation by regulating the microbial community balance. Network analysis provided insights into how bioaugmentation altered microbial interactions. Although the high-temperature bioaugmentation treatment reduced the overall network complexity, it instead strengthened the positive correlations among the remaining taxa.

This shift from competitive to cooperative interspecific relationships, evidenced by increased positive correlation ratios and modularity values, suggests that bioaugmentation promotes synergistic degradation networks. Additionally, compositionality-aware approaches may provide more robust network inference. These methods could be employed in future studies to further extend the findings of this study. Li et al. [55] also found that during the bioaugmentation treatment of oily sludge, exogenous microorganisms improved the bioremediation effect by regulating interspecies relationships. Although high-temperature bioaugmentation reduced the complexity of the network, it significantly strengthened the correlations between the various genera. This approach weakened intergeneric competition, enhanced cooperation, and facilitated the degradation of organic pollutants in the system. An increase from two modules in the blank group to four modules in the bioaugmentation treatment groups was observed. Nodes in different modules perform distinct functions [46]. This phenomenon suggests that bioaugmentation may gradually shift microbial community functions from simplification to diversification. The addition of the surfactant (the T2 group) further enhanced the correlation among the bacteria in the system. Zhuang et al. [56] also observed similar results, revealing that the addition of biosurfactants significantly decreased the negative correlation between bacteria and mitigated competition within the microbial community. Functional prediction analysis showed that the dominant genera possess complementary metabolic capabilities for degrading diverse hydrocarbons including xylene, styrene, and polycyclic aromatic hydrocarbons, with *Paracoccus*, *Nocardioides*, and *Mycobacterium* showing particularly versatile degradation potentials.

**Figure 7:**
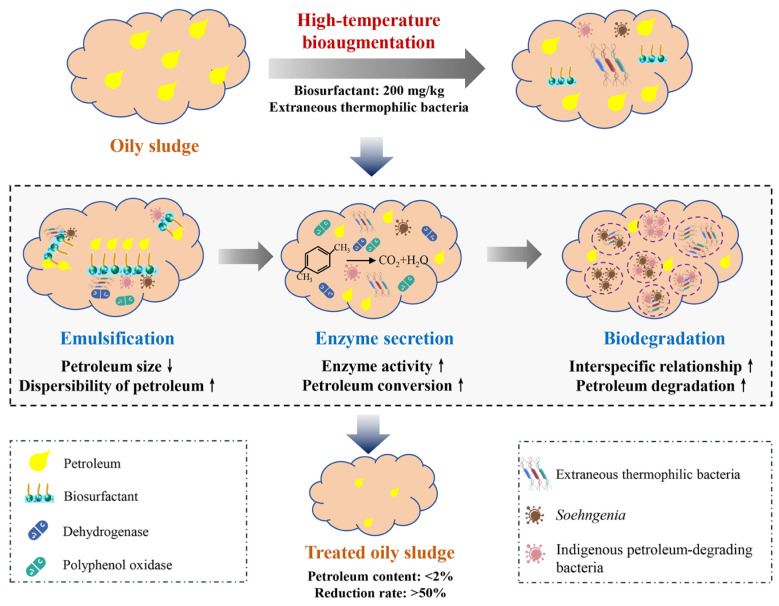
The process of efficient treatment of petroleum pollutants in oily sludge by high-temperature bioaugmentation system.

This study verified the feasibility of oily sludge treatment via high-temperature bioaugmentation and elucidated the mechanisms underlying improved petroleum component degradation, thereby providing a new perspective and a theoretical basis for the bioremediation technology of oily sludge (Figure 7). The bioaugmentation approach primarily involves the introduction of thermophilic petroleum-degrading bacteria and a biosurfactant. Exogenous thermophilic bacteria, combined with indigenous bacteria, formed a powerful petroleum degradation system. First, these biosurfactants can emulsify petroleum droplets into tiny particles, which can be subsequently ingested by petroleum-degrading bacteria and then degraded because of their increased bioavailability in the aqueous phase. Othman et al. [57] also found that the addition of surfactants enhanced the emulsification of petroleum, which provides an abundant substrate for petroleum-degrading bacteria, thereby accelerating petroleum degradation. Second, the rapid growth of exogenous thermophilic petroleum-degrading bacteria under high-temperature conditions promotes the secretion of dehydrogenase and polyphenol oxidase, thereby enhancing the decomposition and conversion of petroleum components, especially in long-chain n-alkanes, PAHs, styrene, naphthalene, and xylene [58]. Meanwhile, indigenous petroleum-degrading bacteria are enriched and interspecific cooperation is enhanced by bioaugmentation. This ecological synergistic effect further enhances the stability and efficiency of the entire bioremediation system and promotes the thorough degradation of petroleum components [59]. This resulted in a low petroleum content in the treated oily sludge (19.6 g/kg), meeting the control standard (SY/T 7301-2016) [29], which fully proves the practicality and environmental friendliness of this technology.

## 5. Conclusions

This study highlights the feasibility of accelerating the degradation of petroleum components in oily sludge using a high-temperature bioaugmentation treatment. Under optimal conditions, the petroleum content was successfully reduced from 60.83 to 19.62 g/kg, meeting the control standard for petroleum content (SY/T 7301-2016). The degradation rate of n-alkanes (C_10_-C_40_) exceeded 70%, while the degradation rate of PAHs exceeded 45%, and the moisture content of the treated oily sludge was less than 5%. High-throughput sequencing results revealed that exogenous thermophilic petroleum-degrading bacteria grew rapidly, became the dominant genera, and enhanced interspecific cooperation among the petroleum-degrading genera, thereby accelerating petroleum degradation. These findings provide a valuable technical option for the rapid treatment of oily sludge.

## Data Availability

The 16S rRNA gene sequences reported in this study were deposited in the GenBank database under accession numbers PQ721805, PQ721806, and PQ721807. The remaining data generated or analyzed during this study were included in this published article and its Supplementary Materials files. All raw paired-end Illumina MiSeq sequences were deposited at the National Center for Biotechnology Information (NCBI; https://www.ncbi.nlm.nih.gov/, accessed on 20 December 2024) under accession number PRJNA1192422.

## Supplementary Materials

The following supporting information can be downloaded at: https://www.mdpi.com/article/10.3390/microorganisms14071470/s1, Figure S1: Appearance morphology of exogenous thermophilic bacteria; Figure S2: High-temperature treatment device; Figure S3: Characterization of the biosurfactant produced by *Brevibacillus agri* AQ-23: critical micelle concentration (a); TLC (b) and FT-IR (c); Figure S4: Effect of biosurfactant dosage on the bioaugmentation treatment; Figure S5: Volatilization ability of petroleum hydrocarbons; Figure S6: Differential bacterial genera at the genus level between different samples. The *x*-axis represents the percentage value of species abundance, the y-axis represents the species name at the genus level, and different colors represent different groups. The *p*-values are shown on the far right, with significance levels indicated by asterisks: * 0.01 < *p* < 0.05, ** 0.001 < *p* < 0.01, *** *p* < 0.001; Figure S7: Microbial co-occurrence network map of the Blank group colored by Phylum level (a) and by module (b). The circular nodes represent bacterial genera, and the thickness of node lines is proportional to the value of Pearson correlation coefficient; Figure S8: Microbial co-occurrence network map of the T1 group colored by Phylum level (a) and by module (b). The circular nodes represent bacterial genera, and the thickness of node lines is proportional to the value of Pearson correlation coefficient; Table S1: The basic physicochemical property of oily sludge after hot washing; Table S2: Design scheme for optimal process conditions of high temperature bioaugmentation; Table S3: Changes in total mass of oily sludge treatment system before and after treatment; Table S4: Alpha diversity index for each treatment; Table S5: Topological properties of the network diagram.

## Abbreviations

The following abbreviations are used in this manuscript:

PAHs	Polycyclic aromatic hydrocarbons
TLC	Thin-layer chromatography
FT-IR	Fourier transform infrared spectroscopy
CMC	Critical micelle concentration
DHA	Dehydrogenase
PPO	Polyphenol oxidase
AD	Average degree
