# Supplementary material for: Insight into Oily Sludge Treatment by High-Temperature Bioaugmentation: Petroleum Components Degradation and Microbial Community Regulation
Source: Microorganisms. 2026 Jul 3;14(7):1470. doi: 10.3390/microorganisms14071470 (PMC13414408; doi:10.3390/microorganisms14071470)
Supplement: Supplementary file 1 [file microorganisms-14-01470-s001.zip › microorganisms-4367466-supplementary.pdf]

Insight into oily sludge treatment by high-temperature bioaugmentation: petroleum components degradation and microbial community regulation

Xinge Fu [1], Jin Li [2,*], Qinghong Wang [2], Xuankai Zeng [2] Hui Zuo [2], Juntao Jiang [2], Rui Zhang [2], Peng Zhao [3], Muhammad Usman [4], Mohamed Gamal El-Din [4,*] and Chunmao Chen [1, 2]

[1] Shandong Key Laboratory of Green Electricity & Hydrogen Science and Technology, School of Chemical Engineering, Shandong Institute of Petroleum and Chemical Technology, Dongying, 257061, China

[2] State Key Laboratory of Heavy Oil Processing, China University of Petroleum-Beijing, Beijing 102249, China

[3] State Key Laboratory of Microbial Diversity & Innovative Utilisation, Institute of Microbiology, Chinese Academy of Sciences, Beijing 100110, China

[4] Department of Civil and Environmental Engineering, University of Alberta, Edmonton, Alberta T6G 1H9, Canada

* Corresponding author:

Jin Li

Phone: +86-010-8973-5022

E-mail: lijincup@163.com

State Key Laboratory of Heavy Oil Processing, Beijing Key Laboratory of Oil and Gas Pollution Control, China University of Petroleum-Beijing, Beijing 102249, China.

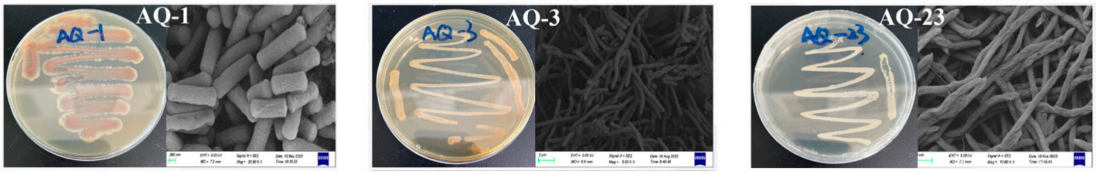

Figure S1 Appearance morphology of exogenous thermophilic bacteria

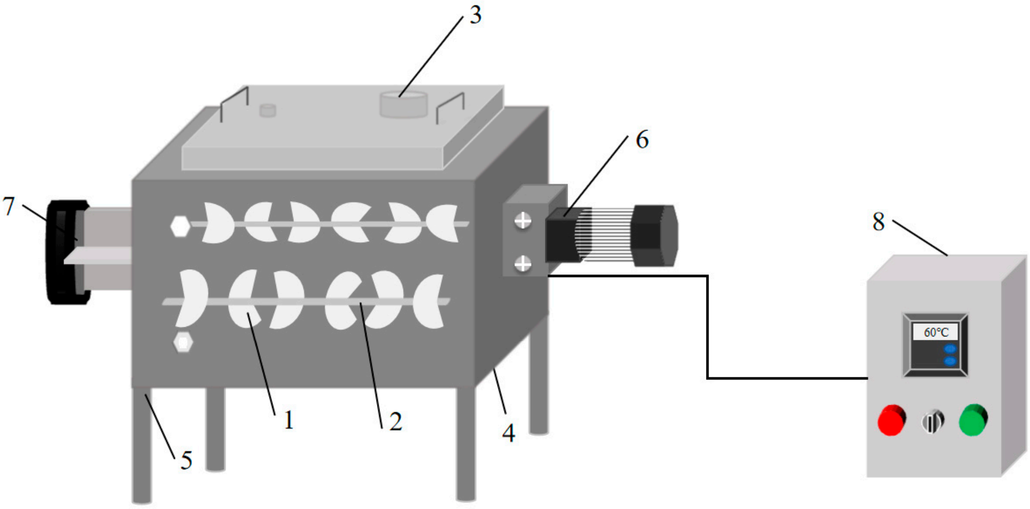

1——Paddle blade; 2——Paddle shaft; 3——Feed inlet; 4——Discharge Outlet; 5——Base; 6——Transmission device; 7——Rotary Joint; 8——Distribution box

Figure S2 High-temperature treatment device

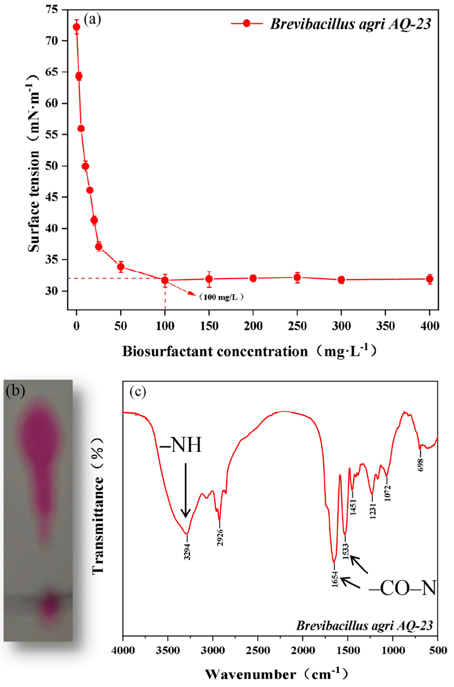

Figure S3 Characterization of the biosurfactant produced by *Brevibacillus agri* AQ-23: critical

micelle concentration (a); TLC (b) and FT-IR (c)

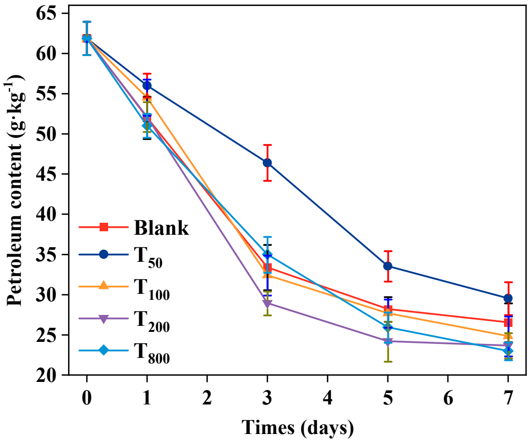

Figure S4 Effect of biosurfactant dosage on the bioaugmentation treatment

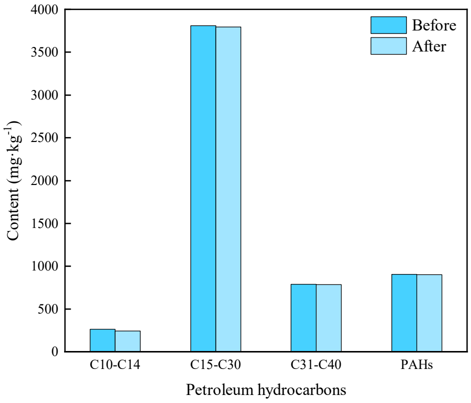

Figure S5 Volatilization ability of petroleum hydrocarbons

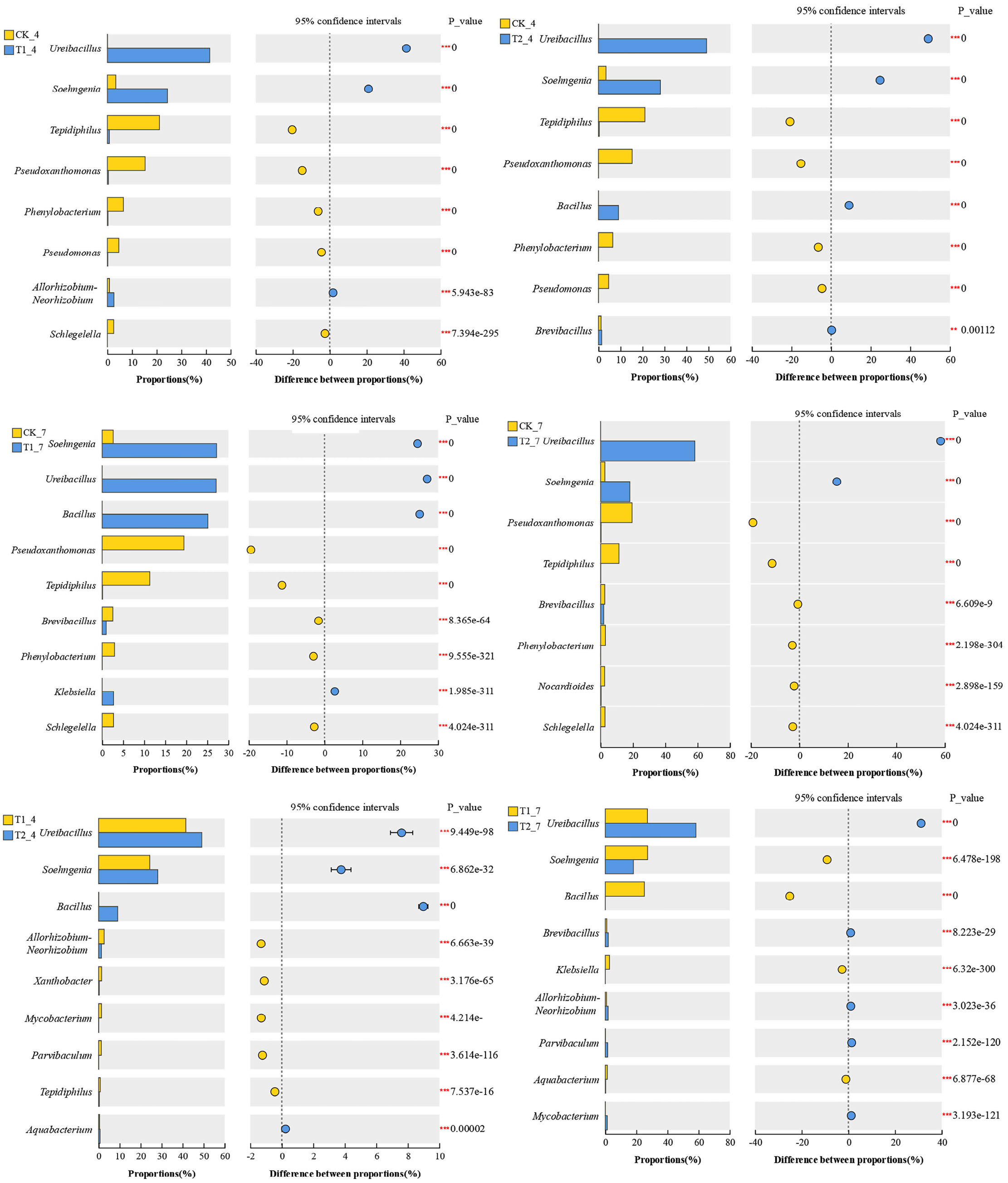

Figure S6 Differential bacterial genera at the genus level between different samples. The x-axis represents the percentage value of species abundance, the y-axis represents the species name at the genus level, and different colors represent different groups. The p-values are shown on the far right, with sig-nificance levels indicated by asterisks: * $0.01 < p < 0.05$, ** $0.001 < p < 0.01$, *** $p < 0.001$

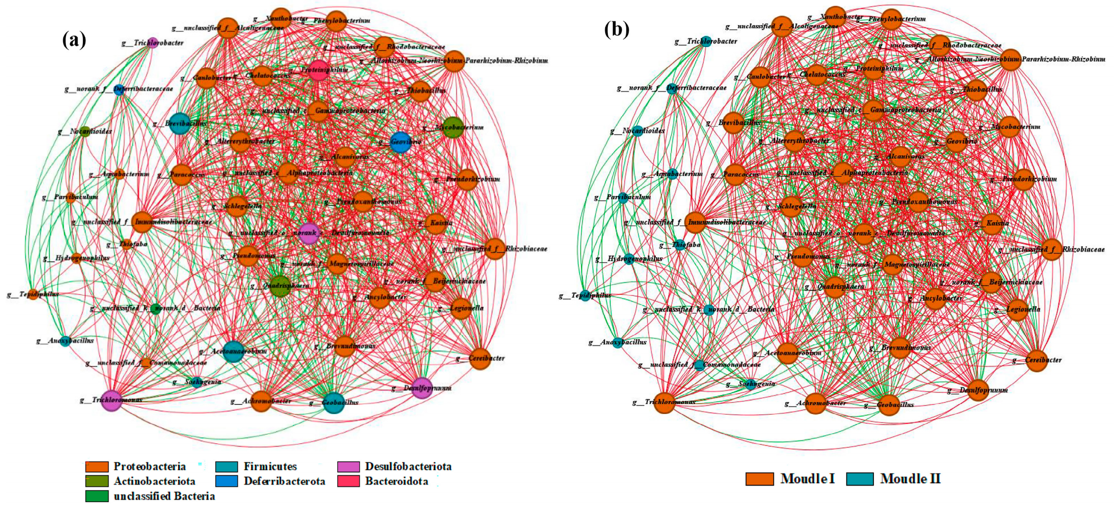

Figure S7 Microbial co-occurrence network map of the Blank group colored by Phylum level (a) and by module (b). The circular nodes represent bacterial genera, and the thickness of node lines is proportional to the value of Pearson correlation coefficient

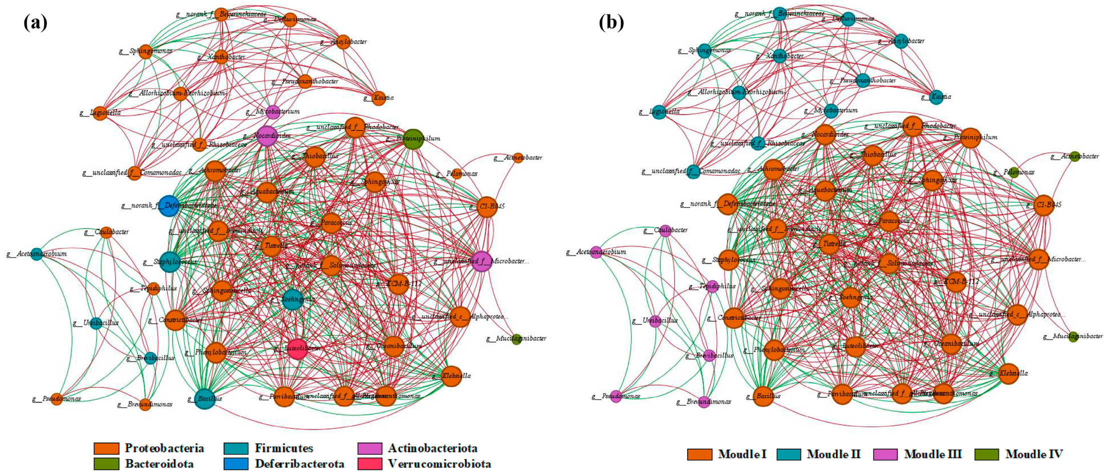

Figure S8 Microbial co-occurrence network map of the T1 group colored by Phylum level (a) and by module (b). The circular nodes represent bacterial genera, and the thickness of node lines is proportional to the value of Pearson correlation coefficient

Table S1 The basic physicochemical property of oily sludge after hot washing

physicochemical property	value
pH	8.25
moisture content (%)	35.66
petroleum content (g/kg)	62.63
organic matter (%)	16.79
total organic carbon (TOC) (%)	12.97
total nitrogen (TN) (%)	0.42
C/N	39.97

Table S2 Design scheme for optimal process conditions of high temperature bioaugmentation

Strain	Drop collapse	Diameter of oil drainage ring (cm)	Emulsification index (%)
Bacillus velezensis AQ-1	+	3.5	40
Ureibacillus thermosphaericus AQ-3	+	3.3	35
Brevibacillus agri AQ-23	++	5.0	55

Note: In the droplet collapse experiment, "+" represents the phenomenon of droplet collapse, while "++" represents a greater degree of collapse.

Table S3 Changes in total mass of oily sludge treatment system before and after treatment

Treatment group	Total sludge mass（g）		Degree of reduction (%)
	Before treatment	After treatment	
CK		2998.69	50.02
T1	6000	2850.09	52.50
T2		2634.16	56.10

Table S4 Alpha diversity index for each treatment

Samples	ACE	Chao	Shannon	Simpson	Coverage	Sobs
D0	968.64	941.84	4.48	0.050	0.9976	922
Blank-4	585.12	583.16	3.43	0.102	0.9998	583
Blank -7	458.74	456.39	2.85	0.194	0.9998	456
T1-4	589.58	576.70	3.56	0.083	0.9984	559
T1-7	242.45	242.40	3.07	0.094	0.9998	240
T2-4	265.87	264.29	2.64	0.155	0.9998	264
T2-7	412.52	406.84	2.93	0.153	0.9995	405

Table S5 Topological properties of the network diagram

Groups	Number of total nodes	Number of total edges	Modularity（MD）	Average degree（AD）
Blank	49	732	0.16	29.88
T1	49	568	0.33	18.72
T2	50	373	0.55	14.92
